# Probiotics, Their Extracellular Vesicles and Infectious Diseases

**DOI:** 10.3389/fmicb.2022.864720

**Published:** 2022-03-30

**Authors:** A. Paula Domínguez Rubio, Cecilia L. D’Antoni, Mariana Piuri, Oscar E. Pérez

**Affiliations:** ^1^Departamento de Química Biológica, Facultad de Ciencias Exactas y Naturales, Universidad de Buenos Aires, Buenos Aires, Argentina; ^2^Instituto de Química Biológica de la Facultad de Ciencias Exactas y Naturales, Universidad de Buenos Aires, Consejo Nacional de Investigaciones Científicas y Técnicas, Buenos Aires, Argentina

**Keywords:** probiotics, GRAS, postbiotics, extracellular vesicles, membrane vesicles, infectious diseases

## Abstract

Probiotics have been shown to be effective against infectious diseases in clinical trials, with either intestinal or extraintestinal health benefits. Even though probiotic effects are strain-specific, some “widespread effects” include: pathogen inhibition, enhancement of barrier integrity and regulation of immune responses. The mechanisms involved in the health benefits of probiotics are not completely understood, but these effects can be mediated, at least in part, by probiotic-derived extracellular vesicles (EVs). However, to date, there are no clinical trials examining probiotic-derived EVs health benefits against infectious diseases. There is still a long way to go to bridge the gap between basic research and clinical practice. This review attempts to summarize the current knowledge about EVs released by probiotic bacteria to understand their possible role in the prevention and/or treatment of infectious diseases. A better understanding of the mechanisms whereby EVs package their cargo and the process involved in communication with host cells (inter-kingdom communication), would allow further advances in this field. In addition, we comment on the potential use and missing knowledge of EVs as therapeutic agents (postbiotics) against infectious diseases. Future research on probiotic-derived EVs is needed to open new avenues for the encapsulation of bioactives inside EVs from GRAS (Generally Regarded as Safe) bacteria. This could be a scientific novelty with applications in functional foods and pharmaceutical industries.

## Introduction

Infectious diseases are disorders caused by organisms such as viruses, bacteria, fungi, or parasites. Could probiotics deal with infectious diseases? A lot of clinical trials have brought this question to the forefront with positive effects on prevention and/or treatment of infectious diseases. In this review, we conducted a search for probiotic bacteria utilized for treatment of infectious diseases and then discussed the current knowledge about extracellular vesicles (EVs) released by these probiotic species. It is important to highlight that according to the generally accepted definition of probiotic, probiotic effects are strain-specific. However, various effects of probiotics can be ascribed to the species level (Hill et al., 2014). Moreover, the study of EVs released by the probiotic strains is still in its infancy and has not been widely analyzed so far. For these two reasons, to collect the current evidence of probiotic-derived EVs we decided to extrapolate our search to the species level. In this line, EV-producing strains were shown to mediate beneficial effects both *in vitro* and *in vivo* models, but human trials (a requirement for probiotic claim) are still pending.

## Probiotics

### Definition

Probiotics are defined as “live microorganisms that, when administered in adequate amounts, confer a health benefit on the host” (Hill et al., 2014). In a position statement an expert panel of the International Scientific Association for Probiotics and Prebiotics (ISAPP) set four minimum criteria for probiotic claims (Binda et al., 2020). Probiotics must: (1) be identified to the genus, species and strain level, (2) be safe for the intended use, (3) have demonstrated health benefits in at least one clinical trial, and (4) have a suitable viable count at end of shelf life.

Before clinical trials are conducted, potential probiotics must be selected by a comprehensive approach including multiple steps. According to the “Guidelines for the Evaluation of Probiotics in Food” (FAO/WHO, 2002), candidate strains are suggested to be assessed for their stress tolerance, antimicrobial properties, epithelium adhesion ability, and safety. At the same time, *in vitro* and *in vivo* experiments should be performed to evaluate probiotic effects (de Melo Pereira et al., 2018; Santos et al., 2020).

As stated above, for validation of treatment safety and efficacy, probiotics must be subjected to at least one clinical trial, which must be conducted based on generally accepted scientific standards (Binda et al., 2020). In general, the weight ascribed to a trial result is higher when sources of bias are avoided (Higgins et al., 2019), and therefore randomized controlled trials are usually considered the most appropriate methodology for validating a probiotic health claim (Tamayo, 2008). In the last decades, there has been a rapid growth in the number of clinical trials for the use of probiotics for prophylactic and/or therapeutic applications in various fields: infectious diseases, cancer, depression and obesity (Zommiti et al., 2020).

Even though probiotics must be identified to the strain level, various meta-analyses indicate that “shared benefits” are achieved by many different strains of the same species, due to similar biological pathways (Sanders et al., 2018). In that regard, the ISAPP panel considered that well-studied beneficial species may be considered as “probiotics” even in the absence of randomized controlled trials that support this claim (Hill et al., 2014). Although clinical trials rarely compare different strains of the same species, certain health effects such as immunomodulation have been ascribed to many strains of the same species (Zhao et al., 2020, 2021).

Many probiotic lactic acid bacteria have long been used in dairy products, being awarded the status of GRAS (Ghosh et al., 2019). The projection of the global probiotics market is expected to grow at a compound annual growth rate of 7.2% from 2021 to 2028 (Grand View Research, 2021). The popularity of probiotic use has increased dramatically in the last decades, not only for their clinical use, but also in healthy individuals wishing to maintain a healthy gut microbiota (Fleming et al., 2016; Su et al., 2020).

### Probiotics and Microbiota: Inter-Kingdom Communication

Our bodies are composed of human cells and microbiota, which is composed of viruses, bacteria, fungi and parasites (Cao and Mortha, 2020). These complex and dynamic populations of microorganisms are crucial for maintaining health and playing a decisive defensive role against pathogens (Sokol, 2019). Inside the human body there exist different microbiotas according to their localization: skin, lung, urethra, vagina, etc. In the last decade, organs that had been previously considered sterile today are hypothesized to have a microbiota. For example, despite it was always thought that the fetus was developed under sterile conditions, recent data suggested the presence of microorganisms in the uterus and placenta (Agostinis et al., 2019; Tang et al., 2020). Moreover, contrary to a long-held dogma, today we know that human milk is not sterile (McGuire and McGuire, 2017). The hypothesis of how bacteria from the maternal gastrointestinal tract (GIT) are translocated to human milk is that dendrites from dendritic cells (DCs) could cross the gut epithelium and transport gut lumen bacteria to the mammary gland through the lymphoid system (Olivares et al., 2015; Demmelmair et al., 2020).

Nowadays, the gut microbiota is considered a new “vital organ” of the human body and is connected with other organs through different axis via neural, endocrine and immune interactions (Ding et al., 2019; Ahlawat et al., 2021). In this line, the consumption of probiotics has been reported to have beneficial effects on the gut–brain axis, the gut–skin axis, etc. (Banfi et al., 2021; Park et al., 2021). In addition, it has recently been demonstrated that the consumption of probiotics can modulate other microbiotas too, e.g., vaginal microbiota (Silvia Ventimiglia et al., 2021).

Fermented foods and probiotics (these terms should not be confused) increase gut microbiota diversity with benefits on human health (Aljutaily et al., 2020; Vinderola and Pérez-Marc, 2021). Frequently, a disruption in the microbiota composition results in a less diverse or less “rich” microbiota, which is often linked to a leaky gut syndrome, higher gut inflammation and more oxidative stress. This microbiota imbalance is linked to various diseases including obesity, diabetes, irritable bowel syndrome, inflammatory bowel disease, depression, and cardiovascular disease (Hills et al., 2019).

It is important to emphasize that many probiotic strains do not colonize the gut and are no longer recoverable in stool 1–4 weeks after stopping their consumption. For example, the probiotic-containing fermented milk Activia did not change the bacterial composition in the gut, but instead altered gene expression patterns that are relevant to carbohydrate metabolism in the gut microbiota. These changes in the gut function were confined only to the time of probiotic consumption (Maguire and Maguire, 2019).

### Probiotics for Infectious Diseases

**FIGURE 1:**
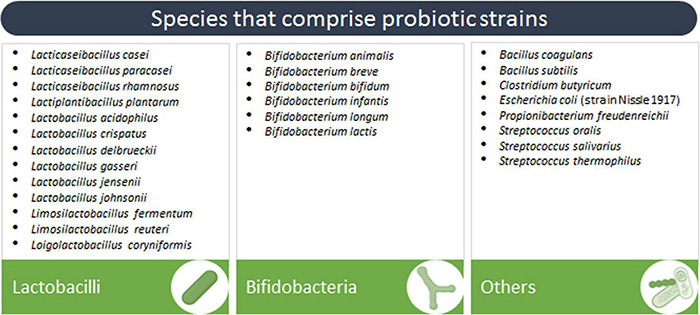
Species that comprise probiotic strains assessed for the prevention or treatment of infectious diseases in clinical trials.

In the context of probiotics against infectious diseases, widespread effects or “shared benefits” of probiotics include mechanisms that act directly by inhibiting pathogens and indirectly by reinforcing the host epithelial barrier function and immune responses (Lebeer et al., 2010; Sassone-Corsi and Raffatellu, 2016; Raheem et al., 2021). Even though probiotic effects are strain-specific, in this review we collected a series of clinical trials where probiotic species benefits were assessed against infectious diseases (Figure 1). According to our search, nearly 50% of these species were reported to release EVs (Table 1). Seven out of 24 strains released EVs that have had beneficial effects against pathogens in *in vitro*, *ex vivo* or *in vivo* models (Table 1, indicated by asterisks). In fact, some of these strains are well-known probiotics: i.e., *Escherichia coli* Nissle 1917 and *Lacticaseibacillus rhamnosus* GG. We limited our search to bacteria, although some fungi are also considered to be probiotics.

**TABLE 1:** Biological effects of EVs released by species that comprise probiotic strains.

Genus and species	Strain	Current evidence	Pathogen inhibition	Barrier function	Immune system	Composition	Transport	Other biological effects	References
*Escherichia coli*	Nissle 1917	EVs improved epithelial barrier function in intestinal epithelial cells (T-84 and Caco-2)		•					Alvarez et al., 2016
*Escherichia coli*	Nissle 1917	EVs protected barrier function in human intestinal epithelial cells (T-84 and Caco-2) infected with *E. coli* (EPEC)		•					Alvarez et al., 2019*
*Escherichia coli*	Nissle 1917	EVs were endocytosed in a clathrin-dependent manner by human intestinal epithelial cells (HT-29)					•		Cañas et al., 2016
*Escherichia coli*	Nissle 1917	EVs incubation with human intestinal epithelial cells (Caco-2) activated NOD1-signaling cascades and NF-κB, and increased IL-6 and IL-8 levels			•				Cañas et al., 2018
*Escherichia coli*	Nissle 1917	EVs increased TNF-α, IL-6, IL-8, IL-10 and MIP1α levels in PBMC, human intestinal epithelial cells (Caco-2)/PMBCs co-culture and *ex vivo* colonic mucosa explants		•	•		•		Fábrega et al., 2016
*Escherichia coli*	Nissle 1917	EVs improved clinical symptoms and histological scores, protected intestinal epithelial barrier function, and mediated anti-inflammatory effects in a dextran sulfate sodium-induced colitis mouse model		•	•				Fábrega et al., 2017
*Escherichia coli*	Nissle 1917	EVs incubation with mouse macrophage cells (RAW264.7) increased TNF-α, IL-4, IL-6, IL-10 and IL-12 levels, and stimulated bacteria-killing ability against *E. coli*, *S. typhimurium, and S. aureus*			•				Hu et al., 2020*
*Escherichia coli*	Nissle 1917	Vaccination with engineered EVs (modified bacteria that express the enterotoxin ClyA) had a strong adjuvant capability on the immune response in mice			•				Rosenthal et al., 2014
*Bacillus subtilis*	168	EVs were transported across human intestinal epithelial cells (Caco-2)					•		Domínguez Rubio et al., 2020
*Bifidobacterium bifidum*	LMG13195	EVs incubation with human dendritic cells induced Treg differentiation and increased IL-10 levels			•				López et al., 2012
*Bifidobacterium longum*	NCC2705	EVs contained several mucin-adhesion proteins				•			Morishita et al., 2021
*Bifidobacterium longum*	–	EVs incubation with mouse macrophage cells (RAW264.7) and dendritic cells (DC2.4) increased TNF-α and IL-6 levels			•	•	•		Morishita et al., 2021
*Clostridium butyricum*	–	EVs incubation with mouse macrophage cells (RAW264.7) and dendritic cells (DC2.4) increased TNF-α and IL-6 levels			•	•	•		Morishita et al., 2021
*Lacticaseibacillus casei*	ATCC 393	EVs contain the protein p75 associated with probiotic effects				•			Dean et al., 2019
*Lacticaseibacillus casei*	ATCC 393	EVs incubation with human intestinal epithelial cells (Caco-2) decreased TLR9 gene expression and IFN-γ levels, and increased IL-4 and IL-10 levels			•				Vargoorani et al., 2020
*Lacticaseibacillus casei*	BL23	EVs contain proteins p40 and p75 associated with probiotic effects				•			Domínguez Rubio et al., 2017
*Lacticaseibacillus casei*	BL23	EVs increased NF-κB levels and induced phosphorylation of epidermal growth factor receptor (EGFR) in human intestinal epithelial cells (HT-29 and T-84, respectively)			•	•			Bäuerl et al., 2020
*Lacticaseibacillus paracasei*	–	EVs decreased NF-κB levels and mRNA levels of TNFα, IL-1α, IL-1β and IL-2, and increased mRNA levels of TGFβ and IL-10 in LPS-induced inflammation in human intestinal epithelial cells (HT-29) and reduce inflammation symptoms of dextran sulfate sodium-induced colitis in mice.			•				Choi et al., 2020
*Lactiplantibacillus plantarum*	APsulloc 331261	EVs increased IL-10, IL-1β and GM-CSF levels in *ex vivo* human skin cultures, and induced monocyte-to-macrophage transition and polarization to M2b in human monocytic cells (THP-1)			•				Kim et al., 2020
*Lactiplantibacillus plantarum*	BGAN8	EVs were endocytosed in a clathrin-dependent manner by human intestinal epithelial cells (HT29)				•	•		Bajic et al., 2020
*Lactiplantibacillus plantarum*	KCTC 11401BP	EVs decreased IL-6 levels and protected cell viability against treatment with *S. aureus* EVs in human epidermal keratinocytes (HaCaT), and reduced skin inflammation in *S. aureus* EV-induced atopic dermatitis in mice			•				Kim et al., 2018*
*Lactiplantibacillus plantarum*	KCTC 11401BP	EVs increased Brain Derived Neurotrophic Factor (BDNF) levels in mouse hippocampal neurons (HT22) and produced antidepressant-like effects in mice with chronic restraint stress						•	Choi et al., 2019
*Lactiplantibacillus plantarum*	WCFS1	EVs prolonged the survival of *C. elegans* infected with vancomycin-resistant enterococci		•		•			Li et al., 2017*
*Lactiplantibacillus plantarum*	WCFS1	EVs incubation with mouse macrophage cells (RAW264.7) and dendritic cells (DC2.4) increased TNF-α and IL-6 levels			•	•	•		Morishita et al., 2021
*Lacticaseibacillus rhamnosus*	GG	EVs decreased TNF-α, IL-1β, IL-6 and MCP-1 levels in LPS-induced inflammation in mouse macrophage cells (RAW264.7), increased IL-22 levels and decreased hepatic bacterial translocation by reinforcing the intestinal barrier function in alcohol-associated liver disease in mice		•	•	•			Gu et al., 2021
*Lacticaseibacillus rhamnosus*	GG	EVs increased apoptosis in human hepatic cells (hepG2)				•			Behzadi et al., 2017
*Lacticaseibacillus rhamnosus*	GG	EVs decreased IFN-γ and IL-17A levels in *S. aureus*-stimulated human PBMC			•				Mata Forsberg et al., 2019*
*Lacticaseibacillus rhamnosus*	GG	EVs inhibited TLR4-NF-κB-NLRP3 axis activation in colonic tissues, and decreased TNF-α, IL-1β, IL-2 and IL-6 levels in dextran sulfate sodium-colitis in mice			•				Tong et al., 2021
*Lacticaseibacillus rhamnosus*	JB-1	EVs increased IL-10 and HO-1 levels via Dectin-1, SIGNR1, TLR-2 and TLR-9 activation in dendritic cells, and increased Treg cells in Peyer’s patch from mice			•				Al-Nedawi et al., 2015
*Lacticaseibacillus rhamnosus*	JB-1	EVs appeared in blood 2.5 h after oral consumption and contained bacteriophage DNA			•	•	•		Champagne-Jorgensen et al., 2021a
*Lacticaseibacillus rhamnosus*	JB-1	EVs were endocytosed in a likely clathrin-dependent manner by mouse (MODE-K) and human intestinal epithelial cells (HT-29) and by mouse intestinal epithelial cells *in vivo*. They expose lipoteichoic acid that activated TLR2 and increased IL-10 levels			•	•	•		Champagne-Jorgensen et al., 2021b
*Lactobacillus acidophilus*	ATCC 53544	EVs contain bacteriocins	•			•			Dean et al., 2019
*Lactobacillus acidophilus*	ATCC 53544	Bacteriocin-enriched EVs fused with other bacteria	•			•			Dean et al., 2020
*Lactobacillus crispatus*	BC3	EVs protected human cervico-vaginal and tonsillar tissues, and human CD4+ T cell lines (MT-4 and Jurkat-tat) from HIV-1 infection by decreasing viral attachment	•			•			Ñahui Palomino et al., 2019*
*Lactobacillus gasseri*	BC12	EVs protected human cervico-vaginal and tonsillar tissues, and human CD4+ T cell lines (MT-4 and Jurkat-tat) from HIV-1 infection by decreasing viral attachment	•			•			Ñahui Palomino et al., 2019*
*Lactobacillus gasseri*	JCM 1131	EVs expose lipoteichoic acid on the surface during logarithmic phase				•			Shiraishi et al., 2018
*Lactobacillus johnsonii*	N6.2	EV expose proteins that are recognized by IgA and IgG from plasma of individuals who had consumed the probiotic			•				Harrison et al., 2021
*Latilactobacillus sakei*	NBRC15893	EVs promoted IgA production by murine Peyer’s patch cells via TLR2			•				Yamasaki-Yashiki et al., 2019
*Limosilactobacillus reuteri*	ATCC 23272	EVs contain no bacteriocins, even though this strain produces high levels of these antibacterial molecules				•			Dean et al., 2019
*Limosilactobacillus reuteri*	BBC3	EVs decreased mRNA levels of TNF-α, IL-1β, IL-6, IL-17 and IL-8, and increased mRNA levels of IL-10 and TGF-β in LPS-induced inflammation in chicken			•	•			Hu et al., 2021
*Limosilactobacillus reuteri*	DSM 17938	EVs decreased IFN-γ and IL-17A levels in *S. aureus*-stimulated human PBMC			•				Mata Forsberg et al., 2019*
*Limosilactobacillus reuteri*	DSM 17938	EVs mimicked the effect of the bacteria on gut motility in mice						•	West et al., 2020
*Propionibacterium freudenreichii*	CIRM-BIA 129	EVs decreased NF-κB and IL-8 levels in LPS-induced inflammation in human intestinal epithelial cells (HT-29)			•	•			Rodovalho et al., 2020

*EVs that have had beneficial effect against pathogens in in vitro, ex vivo, or in vivo models are indicated by asterisks.*

Probiotic bacteria with successful results against infectious diseases mainly include bifidobacteria and lactobacilli, which represent the most studied probiotics (Stavropoulou and Bezirtzoglou, 2020), and other Gram (+) bacteria belonging to the genera *Streptococcus*, *Bacillus*, *Propionibacterium* and *Clostridium.* On the other hand, to our knowledge the only Gram (−) bacterial strain that was found to be effective in clinical trials is *E. coli* Nissle 1917. *E. coli* Nissle 1917 has been considered a probiotic for over a century and used to treat intestinal diseases. However, the strain contains a pathogenicity island (*pks*) that codes for colibactin, a genotoxin that mediates anti-inflammatory effects (Olier et al., 2012) and is now linked to causative mutations found in human colorectal cancer (Nougayrède et al., 2021).

**FIGURE 2:**
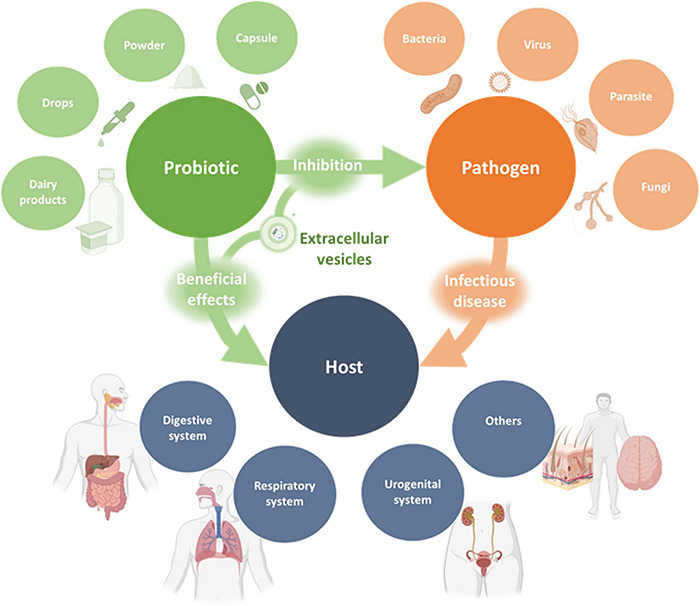
Schematic representation of the interactions between probiotics, pathogens and the host. Probiotics in various dosage forms were shown to exert beneficial effects on different human organ systems for the prevention or treatment of infectious diseases. These effects are exerted indirectly or directly through pathogen inhibition and may be mediated, at least in part, by probiotic-derived EVs.

Probiotics are commonly consumed in food or supplements (Hill et al., 2014; Figure 2). Oral administration, which is the most usual route of administration of probiotics, has resulted in satisfactory outcomes in clinical trials, even when the beneficial effect occurred in extraintestinal sites (Maldonado-Lobón et al., 2015; Panigrahi et al., 2017; Vladareanu et al., 2018; Lazou Ahrén et al., 2021). Possible mechanisms by which oral administration of probiotics may have extraintestinal and systemic effects on the host will be discussed in the following sections. However, there are many possible routes of administration of probiotics, such as mouth rinses and lozenges for periodontal disease (Tsubura et al., 2009; Invernici et al., 2018), vaginal suppositories for trichomoniasis, bacterial vaginosis and recurrent urinary tract infections (Stapleton et al., 2011; Sgibnev and Kremleva, 2020), intranasal administration for upper respiratory tract infections (Passali et al., 2019), and topical application for skin wounds (Peral et al., 2009). In the case of respiratory and skin infections, although topical administration could be advantageous (Lopes et al., 2017; Spacova et al., 2021), their study in clinical trials is currently underrepresented.

It is important to note that many clinical trials examine the use of probiotics as a supplementation to conventional therapy against infectious diseases, such as antibiotic and antifungal agents (Shi et al., 2019; Joseph et al., 2021). In general terms, probiotics have shown effectiveness in preventing infectious diseases in different organ systems, from the respiratory and gastrointestinal tracts to the female urogenital system, among others. As regards gastrointestinal diseases, probiotics were effective in reducing frequency and duration of diarrhea (Francavilla et al., 2012; Park et al., 2017; Sharifi-Rad et al., 2020), reducing symptoms of gastroenteritis and *H. pylori* gastritis (Shafaghi et al., 2016; Shin et al., 2020), and preventing necrotizing enterocolitis (Chang et al., 2017). In respect of respiratory diseases, the benefit of probiotics has been mostly associated with prevention of infections, especially in the upper respiratory tract (Aryayev et al., 2018; Anaya-Loyola et al., 2019; Lazou Ahrén et al., 2021). Finally, certain probiotics were successful in reducing symptoms and frequency of recurrent vulvovaginal candidiasis, bacterial vaginosis and urinary tract infections (Laue et al., 2018; Russo et al., 2019; Sgibnev and Kremleva, 2020), possibly mainly by restoring the normal vaginal microbiota. Other benefits of probiotics demonstrated in clinical trials involve other organ systems, such as skin and the nervous system (Kotzampassi et al., 2015; Xia et al., 2018). Further high-quality clinical trials and meta-analyses should be undertaken to provide stronger evidence for the therapeutic use of probiotics (Stavropoulou and Bezirtzoglou, 2020).

## Extracellular Vesicles

### Bacterial Extracellular Vesicles

Probiotics seem to act through a wide repertoire of mechanisms but the specific pathways and key regulatory molecules underlying their beneficial effects are largely unknown (Plaza-Diaz et al., 2019). In this line, EVs have been associated with diverse functions in cell-to-cell communication and appear to be a common language between kingdoms (i.e., bacteria and eukaryotic cells) (Ñahui Palomino et al., 2021).

Extracellular vesicles are produced by all domains of life: archaea, bacteria and eukarya. To this day it has been seen that EVs appear to be produced by all cell types of all studied organisms. All EVs are composed of a lipid bilayer with membrane proteins and contain DNA, RNA and proteins (Théry et al., 2018). The level of knowledge about bacterial EVs is lower than eukaryotic EVs, but the number of studies is continuously increasing (Ñahui Palomino et al., 2021). In particular, EVs from Gram (+) bacteria have been less studied, and our understanding of their biogenesis and interaction with host cells is just being started (Briaud and Carroll, 2020).

The size of bacterial EVs is in the nanoscale (below 500 nm), and has been related to bacterial physiology including probiotic and pathogenic effects. In the case of Gram (+) bacteria, EVs are called membrane vesicles (MVs) and the lipid bilayer encloses cytosolic material. In contrast, in the case of Gram (−) bacteria, EVs are called outer-membrane vesicles (OMVs) and the lipid bilayer encloses periplasmic material. Gram (+) and Gram (−) bacterial EVs are also different in their surface composition for example the presence of lipopolysaccharide (LPS). The diversity in cargo molecules contained in EVs might explain the variety of described roles ranging from decoys for viral and antibiotic attack, quorum sensing as well as regulation of host immune defense (Kaparakis-Liaskos and Kufer, 2020).

### Postbiotics, a New Concept

As mentioned before, probiotics comprise live microorganisms that confer a health benefit on the host when administered in adequate amounts. At the same time, there is increasing evidence of the health effects of non-viable microorganisms and their bioactive compounds (metabolites that they can produce by fermentation or by their action on food components) (Collado et al., 2019). An expert panel of ISAPP defined a postbiotic as a “preparation of inanimate microorganisms and/or their components that confers a health benefit on the host” (Salminen et al., 2021). In this line, EVs are secretory components associated with probiotic bacteria health benefits and consequently could be considered postbiotics (Wegh et al., 2019).

Extracellular vesicles play a central role in many physiological and pathological processes due to their capacity to transport biologically active macromolecules that can effectively alter the biological properties of target cells. Due to this property, they can be considered novel agents with different therapeutic applications. There are many clinical trials investigating the use of human EVs for various therapeutic approaches, including pathogen vaccination, anti-tumor therapy, regenerative therapies and drug delivery (Lener et al., 2015; Théry et al., 2018). In the case of EVs against infectious diseases there exist two different strategies: evaluation of EVs released naturally by the pathogen or infected cells, and EVs from *in vitro* antigen-pulsed DCs (Wahlund et al., 2017; Riley and Blanton, 2018; Santos and Almeida, 2021). However, to our knowledge there are no clinical trials related to the use of EVs from probiotic bacteria for the prevention and/or treatment of any infectious disease.

## Extracellular Vesicles From Probiotic Bacteria and Infectious Diseases

In order to organize the information, we divided the current evidence of the knowledge about the role of EVs as mediators of probiotic beneficial effects into six categories. The first category addresses the role of EVs against pathogens. The second and third categories are related to their function in the host immune system that can also be divided into three lines of defense: physical and chemical barriers, innate immunity and adaptive immunity. The last categories describe EVs composition, how EVs are uptaken and transported across human cells and other functions.

### Pathogen Inhibition

Probiotics can inhibit pathogens through production of antimicrobial agents and through competitive exclusion of pathogens by competing for adhesion or nutrients in the GIT (Surendran Nair et al., 2017; van Zyl et al., 2020; Raheem et al., 2021).

Antimicrobial agents mainly include reactive oxygen species, lactic acid, and bacteriocins (Rajilić-Stojanović, 2013). Bacteriocins are peptides with antimicrobial activity that have shown to inhibit not only bacteria, but also viruses, fungi and parasites (Dicks and Grobbelaar, 2021; Huang et al., 2021). Furthermore, bacteriocins might be an interesting alternative to the use of antibiotics for infectious diseases caused by antibiotic-resistant bacteria due to their high potency and low toxicity (Cui et al., 2021; Gradisteanu Pircalabioru et al., 2021).

Recent studies show that EVs released by *L*. *acidophilus* ATCC 53544 could deliver bacteriocins and thus kill other bacteria (Dean et al., 2019, 2020). Proteomic analyses revealed bacteriocins are enriched in EVs. Even though bacteriocins investigated by these authors are directed against a *L*. *delbrueckii* strain (Dean et al., 2020), other bacteriocins synthesized by probiotics are able to inhibit or kill pathogens, such as *Listeria monocytogenes*, *Staphylococcus aureus, Acinetobacter baumannii*, *Gardnerella vaginalis*, *Streptococcus agalactiae*, and *Pseudomonas aeruginosa*, in both *in vitro* and *in vivo* models (Gaspar et al., 2018; van Zyl et al., 2019; Hassan et al., 2020). It is noteworthy that EVs may protect bacteriocins from proteases and inactivation molecules that are normally present in the intestine. Whether EVs from probiotics can deliver bacteriocins to pathogens is still unknown and holds great potential for future research.

Several clinical trials have shown that probiotics improved vaginal microbiota composition (Ho et al., 2016; Laue et al., 2018; Vladareanu et al., 2018) and it has been demonstrated that a vaginal microbiota dominated by lactobacilli prevents infections caused by various pathogens, including HIV-1 (Chee et al., 2020). A possible relevant mechanism of EVs related to pathogen inhibition is their ability to prevent pathogen interaction with host cells. It has been demonstrated that some *L. crispatus* and *L. gasseri* EVs reduced HIV-1 attachment to host cells and in this way prevented infection in human cell lines and tissues (Ñahui Palomino et al., 2019). This effect was associated with the reduced accessibility of gp120 (a viral envelope protein) to host target cells after incubating HIV-1 virions with EVs.

Regarding competitive exclusion, probiotics can compete with enteric pathogens for adhesion sites on the mucus layer or on intestinal epithelial cells, and hence prevent pathogen colonization and infection (van Zyl et al., 2020). Competitive exclusion of pathogens has been demonstrated in *in vitro* models (Singh et al., 2017; Tuo et al., 2018), and possibly takes place not only in the GIT but also in the oral cavity and urogenital tract. Numerous authors have investigated the role of pathogenic bacteria EVs in transporting virulence factors and toxins into host cells (Macion et al., 2021). On the other hand, to the best of our knowledge, the only existing report of EVs from probiotics mediating the competition between pathogenic and probiotic bacteria was published by Kim et al. (2018). In this study, it was shown that EVs from *L. plantarum* prevented skin inflammation in a murine model of *S. aureus* EV-induced atopic dermatitis. Concerning the GIT, EVs from probiotics expose adhesion proteins that may interact with the mucus layer and human cells. Although it is likely that this interaction may affect viral and bacterial attachment, there remains a need for *in vitro* and *in vivo* studies addressing this question.

### Barrier Function: Physical and Chemical Defense

The intestinal epithelial barrier acts as the first line of defense by avoiding the entrance of antigens and pathogens (Barbara et al., 2021). The alteration of the gut microbiota is the most important factor that disrupts the integrity of the intestinal epithelial barrier, leading to intestinal inflammation and diseases (Gareau et al., 2010). Probiotics, in this context, as transient constituents of the microbiota, are able to improve barrier function by surface components and secreted factors (postbiotics), among them, EVs (Liu et al., 2020). Since the exposure to infection can lead to the loss of epithelial integrity (König et al., 2016; Invernizzi et al., 2020), probiotic EVs participation in the improvement of barrier function could be an important point to regard them as potential prophylactic or therapeutic agents against infections.

As for the physical barrier, *in vitro* and *in vivo* experiments have demonstrated that EVs released by *E. coli* Nissle 1917 can mediate anti-inflammatory effects and protect the intestinal epithelial barrier function (Alvarez et al., 2016; Fábrega et al., 2016, 2017). A key role in the maintenance of intestinal epithelial barrier integrity is played by tight junctions, which are composed of a network of proteins that regulate paracellular permeability, such as claudins, zonula occludens (ZO) and occludin (Barbara et al., 2021). EVs released by *E. coli* Nissle 1917 have been shown to upregulate ZO-1 and claudin-14, downregulate claudin-2 (a gene that codes for a leaky protein), and in turn improve epithelial barrier function in an *in vitro* intestinal epithelium models (T-84 and Caco-2 cell lines) (Alvarez et al., 2016). This function of EVs has also been reported in these same cell lines infected with enteropathogenic *E. coli* (EPEC), an enteric pathogen that disrupts tight junctions as a way to increase invasion. In this work, EVs released by *E. coli* Nissle 1917 were able to counteract EPEC-altered mRNA levels of claudin-14 and occludin, preserve subcellular localization of ZO-1 and occludin, and maintain F-actin at the intercellular junctions. Barrier integrity restoration was further confirmed by measuring transepithelial electrical resistance (TEER) and the flux of FITC-dextran (Alvarez et al., 2019). In addition, restoration of epithelial integrity by EVs has been observed in an *in vivo* model of experimental colitis (Fábrega et al., 2017). In this regard, these authors demonstrated that oral administration of EVs from *E. coli* Nissle 1917 increased: trefoil factor 3 (TFF-3) mRNA levels, a marker of intestinal barrier function; and decreased MMP-9 mRNA levels, a protein involved in tissue injury.

Regarding the intestinal chemical defense, antimicrobial peptides and the mucus layer (mainly produced by the goblet cells) are further key factors that maintain intestinal barrier integrity by protecting epithelial cells from bacteria and other challenges (Hansson, 2020; Yong et al., 2020; Barbara et al., 2021; Fusco et al., 2021). In an *in vivo* model of experimental colitis, treatment with EVs from *E. coli* Nissle 1917 resulted in the restoration of the mucin content in goblet cells and in a smaller ulceration surface, as evidence of barrier integrity (Fábrega et al., 2017). On the other hand, a recent study conducted by Gu and colleagues showed that EVs from *L. rhamnosus* GG increased nuclear factor erythroid 2-related factor 2 (Nrf2) expression and, in turn, increased tight junction proteins and antimicrobial peptide Reg3 levels, which is involved in the prevention of *Listeria monocytogenes* and *Salmonella enteritidis* infections (Loonen et al., 2014; Gu et al., 2021). Furthermore, mRNA Reg3 levels increased after incubation of Caco-2 cells with EVs from *L. plantarum* WCFS1 (Li et al., 2017). In an *in vivo* model, the administration of these EVs prolonged the survival of *Caenorhabditis elegans* infected with vancomycin-resistant enterococci. In this line, EV-mediated protection against antimicrobial resistant pathogens could be useful to limit the development of antibiotic resistance that results from the widespread use of antibiotics.

### Innate and Adaptive Immunity

As mentioned before, intestinal epithelial cells provide a physical barrier that separates the host from the external environment and form not merely static physical barriers: on the contrary, intestinal epithelial cells engage in a complex dynamic crosstalk between the microbiota and the intestinal immune system (Takiishi et al., 2017). Both bacteria and host-derived EVs are key players of such inter-kingdom crosstalk. There is now an accumulating body of evidence that bacterial EVs regulate the innate and adaptive immune system of the host. Consequently, EVs released by the gut microbiota may have great influence on human health and disease. EVs also carry a set of molecules known as microbe-associated molecular patterns (MAMPs) that are recognized by specific receptors expressed by host epithelial and immune cells. These pattern recognition receptors (PRRs), such as TLR2 and NOD1, are key components of innate immunity and mediate host responses (Lebeer et al., 2010; Díaz-Garrido et al., 2021).

Maintaining the proper balance of immune responses at mucosal surfaces is critical for maintaining homeostasis and successfully clearing pathogens. Epithelial cells have been identified as key players in the development of elaborate immune responses that discriminate between non-pathogenic and pathogenic microorganisms. In this regard, intestinal epithelial cells contribute to delaying and dampening infections by initiating the development of an immune response and attracting immune cells to the infectious site (Pellon et al., 2020). Among intestinal epithelial cells, enterocytes are the most abundant cells and represent approximately 90% of the total number. To study absorption and immune responses there are different *in vitro* models of cell lines: Caco-2, HT-29, and T-84. The remaining 10% of the cells consist of mucus-producing goblet cells, enteroendocrine cells, antimicrobial peptide-producing Paneth cells and others (Jochems et al., 2018).

*In vitro* and *in vivo* experiments with intestinal epithelial cells have demonstrated that EVs released by *E. coli*, *L. casei*, *L. paracasei*, *P. freudenreichii*, and *L. rhamnosus* can modulate NFκB levels (Cañas et al., 2018; Bäuerl et al., 2020; Choi et al., 2020; Vargoorani et al., 2020; Tong et al., 2021). NF-κB is a family of transcription factors and has an essential role in a variety of aspects related with human health including the development of both innate and adaptive immunity. EVs from *L. paracasei* and *P. freudenreichii* decreased NF-κB levels in LPS-induced inflammation in HT-29 cell line (Choi et al., 2020; Rodovalho et al., 2020). At the same time, *L. rhamnosus* EVs had the same effect in an *in vivo* model of dextran sulfate sodium-induced colitis in mice (Tong et al., 2021). On the other hand, EVs from *E. coli* and *L. casei* increased NF-κB levels *per se* in Caco-2 and HT-29 cell lines (Cañas et al., 2018; Bäuerl et al., 2020). This opposite modulation of NFκB levels, in the presence or absence of LPS, was also observed for pro-inflammatory cytokines like IL-8 in both Caco-2 and HT-29 cell lines, and in *ex vivo* human colonic explant (Fábrega et al., 2016; Choi et al., 2020; Vargoorani et al., 2020).

On the contrary, in the presence or absence of LPS, *L. casei* and *L. paracasei* EVs always increase the levels of anti-inflammatory cytokines like IL-10. The inhibition of the NF-κB pathway and the increase of IL-10 by EVs have been extensively reported for probiotic bacteria in both *in vitro* and *in vivo* models of infection and/or inflammation (Liu et al., 2017; Bhardwaj et al., 2020). Moreover, Fábrega and colleagues demonstrated in an *in vivo* model of dextran sulfate sodium-induced colitis in mice that *E. coli* Nissle 1917 EVs decreased COX-2 and iNOS mRNA levels that encode important inducible enzymes for the synthesis of prostaglandins and nitric oxide, respectively. This decrease in COX-2 and iNOS levels leads to inflammation and tissue damage, and correlated with the reduced expression of the pro-inflammatory cytokines TNF-and IFN-γ, lower colon inflammation and tissue damage in EV-treated mice (Fábrega et al., 2016, 2017). This evidence suggests that EVs could mediate, at least in part, the beneficial effect of probiotics against infectious diseases.

With regard to immune cells, EVs from different species increase *per se* the levels of pro-inflammatory cytokines like TNF-alpha and IL-6 (Fábrega et al., 2016; Hu et al., 2020; Gu et al., 2021; Morishita et al., 2021) and, at the same time, increase the level of anti-inflammatory cytokines like IL-10 and IL-22 produced by macrophages, DCs and peripheral blood mononuclear cells (PBMC) (López et al., 2012; Al-Nedawi et al., 2015; Fábrega et al., 2016; Hu et al., 2020). In agreement with this effect, it has been reported that different probiotic bacteria stimulate pro-inflammatory and/or anti-inflammatory cytokines in different immune cells (Ren et al., 2016; Cristofori et al., 2021).

Modulation of the immune system by bacterial EVs has also been studied against pathogens in *in vitro* models. EVs from *L. rhamnosus* GG and *L. reuteri* DSM 17938 decreased inflammatory mediators like IFN-γ and IL-17A in *S. aureus*-stimulated human PBMC (Mata Forsberg et al., 2019), while EVs from the probiotic strain *E. coli* Nissle 1917 improved the antibacterial activity of macrophages against three bacterial pathogenic strains of *E. coli*, *S. typhimurium*, and *S. aureus* (Hu et al., 2020).

Regarding macrophage differentiation, *L. plantarum* APsulloc 331261 EVs induced monocyte-to-macrophage transition and polarization to M2b in human THP-1 (Kim et al., 2020). M2b, a subtype of M2 macrophages, has attracted increasing attention due to its strong immunoregulatory and anti-inflammatory effect (Wang et al., 2019). Probiotic bacteria are reported to have a beneficial effect on the host immune status through their ability to modulate macrophage polarization. Some probiotic strains are reported to activate macrophages to M1 phenotype to kill intracellular pathogens, while some other probiotics can induce M2 macrophages to exert an anti-inflammatory effect. Similarly, another strain of the same species (*L. plantarum* CLP-0611) also ameliorated colitis in mice by polarizing M1 to M2-like mouse peritoneal macrophages (Jang et al., 2014).

In line with the anti-inflammatory effects of bacterial EVs, *L. paracasei* and *L. reuteri* BBC3 EVs increased mRNA levels of TGF-β in a model of LPS-induced inflammation in human intestinal epithelial cells (HT-29) and jejunum tissues from chicken (Choi et al., 2020; Hu et al., 2020). TGF-β plays a critical role in the development of Treg cells (Zhao et al., 2017). At the same time, *B. bifidum* LMG13195 and *L. rhamnosus* JB-1 EVs incubation with human DCs induced differentiation to Treg cells and increased IL-10 levels (López et al., 2012), and *L. rhamnosus* EVs increased the number of Treg cells in Peyer’s patch from mice (Al-Nedawi et al., 2015). While in some instances Treg cells appear to limit the efficiency of antiviral protective immunity, in other cases they reduce the level of tissue damage caused by a virus infection (Veiga-Parga et al., 2013).

Regarding adaptive immunity, vaccination with engineered EVs from the probiotic strain *E. coli* Nissle 1917 in mice increased the levels of IgG against a recombinant antigen comparable to the “gold standard” adjuvant (alum) (Rosenthal et al., 2014). This strong adjuvant capability of EVs from probiotic strains provides evidence that engineered EVs could be a useful platform for vaccines in humans. On the other hand, it is interesting to note that *L. johnsonii* N6.2 EVs are recognized by IgA and IgG from the plasma of individuals who had consumed the probiotic. In particular, the increase of IgA occurs as a result of a specific response to EV components: Sdp_SH3b2 and Sdp_SH3b6 (Harrison et al., 2021). Although the function of bacterial SH3b domains is not completely known, they are proposed to be cell wall binding domains in prokaryotes. In a previous work, the authors had shown that *L. johnsonii* N6.2 increased circulating levels of IgA (Marcial et al., 2017). Moreover, it has been demonstrated that *L. sakei* EVs enhanced IgA production by murine Peyer’s patch cells (Yamasaki-Yashiki et al., 2019). A similar study found that commensal bacteria increase the serum levels of IgA, providing a protective effect against polymicrobial sepsis (Wilmore et al., 2018). Therefore, serum IgA concentrations depend on the interaction with the gut microbiota and these effects could be mediated, at least in part, by EVs.

It is interesting to note that *L. plantarum* KCTC 11401BP EVs decreased IL-6 levels, protected cell viability of human epidermal keratinocytes (HaCaT) incubated with *S. aureus* EVs and reduced skin inflammation in *S. aureus* EV-induced atopic dermatitis in mice (Kim et al., 2018). Moreover, *L. plantarum* EVs increased IL-10 and granulocyte Macrophage Colony-Stimulating Factor (GM-CSF) levels in *ex vivo* human skin cultures (Kim et al., 2020). These findings suggest that oral administration of bacteria could have a preventive effect on skin inflammation and these effects could be mediated by EVs. As mentioned below in Section “Uptake and Transport,” *L. rhamnosus* JB-1 EVs appeared in blood after oral consumption and consequently the presence of EVs in the bloodstream could in part explain the benefit of probiotics in extraintestinal tissues and organs (Stentz et al., 2018; Champagne-Jorgensen et al., 2021a).

### Composition

Throughout the years, it has been shown that the supernatant from probiotic bacteria exert beneficial effects in both *in vitro* and *in vivo* models (De Marco et al., 2018; Mantziari et al., 2020). For instance, the culture supernatant from *L. rhamnosus* GG induces resistance to *Escherichia coli* K1 infection by enhancing intestinal defense in neonatal rats (He et al., 2017). In recent years, with the discovery of EVs from probiotics, we can speculate that at least part of these beneficial effects could be mediated by EV components.

As far as we know, there are differences in EV metabolite, nucleic acid and protein content compared with that of the bacterial cell (Briaud and Carroll, 2020). The relative abundance of certain components suggests not only a possible sorting mechanism to package EV cargo, but also a special biological role for EVs (Kim et al., 2018; Huang et al., 2021). For example, EVs from *L. rhamnosus* GG contain high levels of tryptophan metabolites that lead to an improved barrier function (Gu et al., 2021).

In the last few years, “omics” approaches, such as proteomics, transcriptomics and metabolomics, have enabled a comprehensive characterization of probiotics and their EVs, allowing us to gain a deeper understanding of their mechanisms of action (Cunningham et al., 2021). Proteomic analyses showed that EVs from *Lacticaseibacillus* genus (including *L. casei* and *L. rhamnosus* species) contain p40 and p75, two proteins associated with probiotic effects (Domínguez Rubio et al., 2017; Dean et al., 2019; Gu et al., 2021). In particular, p40, when administered in early life, increased TGF-β levels in mice and consequently prevented intestinal inflammation in adulthood (Gu et al., 2021). These proteins, p40 and p75, are able to induce the phosphorylation of the epidermal growth factor receptor (EGFR), and thus have anti-apoptotic effects, as demonstrated in intestinal epithelial cells (Zhang et al., 2020). For p40, this effect was also observed in a murine model of colitis (Yan et al., 2011). EGFR activation can also be triggered by *L. casei* EVs, which expose p40 and p75 at the surface (Bäuerl et al., 2020). Intriguingly, EVs from *L. rhamnosus* GG were shown to have apoptotic effects in hepatic cancer cells by the intrinsic pathway of apoptosis (Behzadi et al., 2017). Therefore, apoptotic effects seem to depend on the dose of EVs and on the model used. On the other hand, p40 and p75 were able to prevent the disruption of tight junctions by protein kinase C (PKC)-dependent mechanisms in Caco-2 cell monolayers (Seth et al., 2008). Anti-apoptotic effects and protection of tight junctions in intestinal epithelial cells are related to an enhancement of intestinal epithelial integrity, a key factor in the maintenance of barrier function, the first line of defense. On the other hand, p40 was proven to increase IgA levels. As mentioned in Section “Innate and Adaptive Immunity,” IgA further contributes to the protection of the host against infections (Ho et al., 2016; Wang and Jeffery, 2016).

It has been shown that EVs from probiotics contain proteins that could mediate pathogen inhibition, and in this way could possibly compete with pathogens for colonization in the intestine (Domínguez Rubio et al., 2017; Bajic et al., 2020; Bäuerl et al., 2020; Nishiyama et al., 2020). Proteomic analyses of EVs from three different lactobacilli strains showed that protein composition of EVs can be very different among species (Dean et al., 2019). Interestingly, antimicrobial bacteriocins are enriched in EVs from *L. acidophilus* ATCC 53544. These EVs can fuse with other bacteria and thus may constitute a useful platform for the delivery of antimicrobial compounds (Dean et al., 2020). On the other hand, it would be interesting to investigate the occurrence of moonlighting proteins in EVs. Moonlighting proteins are proteins that have different functions according to their cellular location (Wang and Jeffery, 2016; Jeffery, 2018). For example, glyceraldehyde-3-phosphate dehydrogenase (GAPDH), a well-known cytoplasmic metabolic protein, is exposed at the surface of the bacterial cell and performs adhesion functions. Further analyses are necessary to confirm the localization of these proteins within EVs to better understand their multiple functions.

Indeed, EV composition is relevant to understand their biological function, even in the context of infections. EVs from *L. crispatus* BC3 and *L. gasseri* BC12, but not EVs from other strains, were capable of protecting vaginal tissues from HIV-1 infection *ex vivo*, suggesting virus inhibition was due to the presence of specific components of EVs (Ñahui Palomino et al., 2019). Regarding immunomodulatory effects of EVs from probiotics, EVs from *Propionibacterium freudenreichii* contain surface-layer protein B (SlpB), which effectively mitigated NF-κB activation (Rodovalho et al., 2020).

Lipoteichoic acid (LTA) has been found on the surface of EVs from *L. gasseri* JCM 1131, *L. casei* BL23 and *L. rhamnosus* JB-1 (Shiraishi et al., 2018; Champagne-Jorgensen et al., 2021b). LTA is a ligand for TLR2 in a heterodimer with TLR6, and it seems to induce immune tolerance in intestinal epithelial cells (Lebeer et al., 2010). In agreement with this, EVs from *L. rhamnosus* JB-1 expose LTA, which was responsible for TLR-2 activation and increase of IL-10 production by bone marrow-derived DCs (Champagne-Jorgensen et al., 2021b). LTA from probiotics could play a role in attenuating infections. In this regard, it has been shown that *L. plantarum* LTA inhibits virus-induced inflammatory responses in porcine intestinal epithelial cells and reduced *Enterococcus faecalis* biofilm *in vitro* (Kim et al., 2017, 2020).

As mentioned before, peptidoglycan contained in EVs from Gram (+) and Gram (−) probiotics is also an important factor in the enhancement of innate immunity and the maintenance of intestinal homeostasis (Cañas et al., 2018; Morishita et al., 2021). In fact, EVs from *Bifidobacterium longum*, *Clostridium butyricum*, and *L. plantarum* WCFS1 have been proposed as a novel immunotherapy formulation that would be advantageous over bacterial lysates due to protection from degradation of bioactives within EVs (Morishita et al., 2021).

As aforesaid, EVs from the Gram (−) probiotic strain *E. coli* Nissle 1917 were shown to have a strong adjuvant capability. The authors ascribed this result to LPS, proteins and glycosyl composition (Rosenthal et al., 2014). The presence of LPS and other MAMPs, such as flagellin and mannose, may be responsible for the strong immune response when applying these EVs as vaccine platforms.

Previous work has established that bacterial EVs contain DNA and RNA (Koeppen et al., 2016; Bitto et al., 2017; Li and Stanton, 2021). Regarding EVs from probiotics, little is known about their nucleic acid cargo. Even though DNA and RNA were found in EVs from *L. reuteri* BBC3 and *L. casei* BL23 (Domínguez Rubio et al., 2017; Hu et al., 2021), the characterization of nucleic acids from probiotics remains to be studied. Small RNA contained in EVs from probiotics might possibly regulate gene expression in host cells, as it is the case for EVs from pathogenic bacteria, and this interaction could have implications in preventing and treating infections (Lee, 2019; Munhoz da Rocha et al., 2020).

Extracellular vesicles from probiotics have shown to contain phage nucleic acids (Domínguez Rubio et al., 2017; Champagne-Jorgensen et al., 2021b; Gu et al., 2021) and phage proteins (Domínguez Rubio et al., 2017; Gu et al., 2021). EVs can even transmit phage receptors to phage-resistant bacteria, which in turn become phage-sensitive (Tzipilevich et al., 2017). Both phage nucleic acid and phage-receptors transmission would lead to a broadened phage host range with potential applications in the treatment of infections (Liu et al., 2018).

### Uptake and Transport

The communication between bacteria and the host could in part occur through bacterial EVs and other soluble factors (postbiotics). EVs are able to transport diverse bioactive molecules to host cells and trigger different effects such as the modulation of immune responses. It is generally accepted that, due to their nanosize, bacterial EVs can overcome epithelial barriers and migrate long distances in the human body (Macion et al., 2021). In fact, bacterial EVs have been demonstrated to enter host cells by several routes, including clathrin, caveolin or lipid raft mediated endocytosis, and membrane fusion (O’Donoghue and Krachler, 2016). Even though much research in the last decades has focused on the study of uptake and transport of EVs released by pathogenic bacteria (Bielaszewska et al., 2017; Bitto et al., 2017), few researchers have addressed these issues for EVs released by non-pathogenic bacteria. However, in the last years there has been an increase in research trying to understand the way that EVs from probiotics are internalized by host cells, or even more, transported through the intestinal barrier and delivered to different tissues and organs.

Before being uptaken by intestinal cells, EVs must also diffuse through the mucus layer. In this regard, EVs from *E. coli* Nissle 1917 were able to diffuse through the mucus layer in the mucin-producer HT29-MTX cell line (Cañas et al., 2016). Although there is no direct evidence of EV diffusion through the mucus layer *in vivo*, this event can be assumed from the fact that EVs can reach the bloodstream after oral administration (Champagne-Jorgensen et al., 2021a).

Extracellular vesicles from probiotics were proven to be internalized by intestinal epithelial cells in several studies (Fábrega et al., 2016; Bajic et al., 2020; Domínguez Rubio et al., 2020; Champagne-Jorgensen et al., 2021b). Although there are several routes of entry for EVs from pathogens into epithelial cells, clathrin-mediated endocytosis has been the most widely reported route among EVs from probiotics so far. Inhibitors of clathrin-mediated endocytosis, such as chlorpromazine and the dynamin dynasore, blocked the uptake of EVs by intestinal cells cultivated *in vitro* (Cañas et al., 2016; Bajic et al., 2020; Champagne-Jorgensen et al., 2021b). Additionally, EVs from *L. rhamnosus* JB-1 were shown to be internalized by intestinal epithelial cells in an *in vivo* model within 2 h after oral consumption (Champagne-Jorgensen et al., 2021a). It is likely that EVs are internalized simultaneously by different endocytic pathways depending on their size (El-Sayed and Harashima, 2013).

With respect to intracellular trafficking, colocalization analyses showed that EVs from *E. coli* Nissle 1917 are present in early endosomes and, once inside the cell, EV peptidoglycan interacts with NOD1 that leads to the activation of the immune system (Cañas et al., 2016, 2018; Fernández-García et al., 2021). Moreover, EVs can also fuse with lysosomes (Cañas et al., 2016). On the other hand, it was demonstrated that EVs from a pathogenic *E. coli* strain can deliver toxins to other subcellular compartments including the cytosol, nucleus and mitochondria (Bielaszewska et al., 2017). Bacterial EVs can deliver DNA or RNA to host cells (Bitto et al., 2017; Lécrivain and Beckmann, 2020), and there is evidence that nucleic acid cargo of EVs from pathogens may enter the nucleus of eukaryotic host cells (Blenkiron et al., 2016; Bitto et al., 2017). Furthermore, EVs from pathogens contain small RNA that might regulate gene expression in host cells (Koeppen et al., 2016). Although little studied to date, these mechanisms may be also applicable to EVs from probiotics.

While a portion of EVs may act in intestinal cells, another portion is possibly transported through the intestinal epithelium, either by paracellular or transcellular transport, to finally reach extraintestinal tissues and organs (Jang et al., 2015; Stentz et al., 2018; Jones et al., 2020). Park et al. (2017) revealed EVs from intestinal bacteria reach the bloodstream in a mouse model, where blood EV diversity was directly linked to intestinal microbiota diversity. Regarding probiotics, a proportion of CFSE-labeled EVs from *Bacillus subtilis* were transported through a monolayer of polarized epithelial cells in a transwell system. Transcellular transport resulted in the detection of intact EVs in the lower chamber in 60–120 min (Domínguez Rubio et al., 2020). Alternatively, EVs could possibly be transported through the intestinal epithelium via DCs, goblet cells or M-cells. On the other hand, microbiota EV transport through the epithelium can occur by paracellular transport when the intestinal epithelial barrier integrity is compromised (Chronopoulos and Kalluri, 2020).

The transport of EVs across the intestinal epithelium implies that EVs could reach the lamina propria, where they are able to interact with immune cells. EV uptake by immune cells has been described in a few studies. *In vivo* studies showed that EVs from *L. rhamnosus* JB-1 were uptaken by DCs in the lamina propria (Champagne-Jorgensen et al., 2021b). This internalization was thought to occur via clathrin-mediated endocytosis, as it was prevented by dynasore, even though phagocytosis cannot be ruled out since dynamin is required for this process. In another study, probiotic-derived EVs were uptaken by mouse macrophage-like and DCs via clathrin-mediated endocytosis and macropinocytosis, as demonstrated in the presence of endocytosis inhibitors (Morishita et al., 2021).

Different studies support EVs distribution and delivery to distal body sites. For example, EVs from *L. rhamnosus* JB-1 were present in the bloodstream of mice fed with the bacteria (Champagne-Jorgensen et al., 2021a), as demonstrated by the detection of DNA from prophages in EVs. What is more, oral administration of EVs from *L. plantarum* reduced skin inflammation in mice with *S. aureus* EV-induced atopic dermatitis (Kim et al., 2018). In humans, microbiota-derived EVs were able to reach urine. In fact, urine-EVs were proposed as a useful assessment method of microbiota profiles (Li et al., 2017). In another study, intraperitoneally injected EVs from *L. plantarum* increased brain-derived neurotrophic factor (BDNF) mRNA levels in the hippocampus of mice and produced antidepressant effects (Choi et al., 2019). This increase in gene expression in the brain suggests that EVs might possibly cross the brain blood barrier. Indeed, EV transport could be one of the reasons why probiotics consumption exerts not only local but also systemic effects, since it is likely that EVs are released by probiotics in the GIT after the consumption of these bacteria.

### Other Biological Effects

As it can be inferred from probiotic beneficial effects, modulation of symptoms is one important factor that explains the clinical efficacy of probiotics in the treatment of infectious diseases. It is often observed that EVs mimic the effect of the parent bacteria. For example, *L. reuteri* DSM 17938 clinical efficacy has been demonstrated for the treatment of colic, diarrhea and constipation (Coccorullo et al., 2010; Chau et al., 2015; Dinleyici et al., 2015). Accordingly, EVs from this strain could reproduce the bacteria beneficial effects on gut motility in jejunum and colon explants from mice (West et al., 2020). Therefore, EV release could be one mechanism whereby probiotics mediate their beneficial effects.

In relation to stress and immunity, chronic stress leads to constantly high corticosteroid levels in blood, an impaired immune function, and an increased susceptibility to infections and other health disorders (Bae et al., 2019). At the same time, exposure to stress can cause a decrease in the expression of BDNF in humans, a molecule with antidepressant-like effects (Yang et al., 2015). Some probiotics were shown to be antidepressants in patients and animal models, and even though the gut–brain axis is involved in this effect, the mechanisms of action are not completely understood (Yong et al., 2020). EVs might come into play here. In this regard, EVs from *L. plantarum* KCTC 11401BP counteracted the decreased levels of BDNF mRNA in the hippocampus of corticosteroid treated mice and also blocked the decrease in the levels of BDNF mRNA in corticosteroid post-treated mice, which was further evidenced in mice antidepressant behavior (Choi et al., 2019). If the anti-depressant effects of EVs are proven, they could possibly participate in preventing and/or treating infections given that immune function may be impaired in patients with depression (Andersson et al., 2016).

## Discussion

### Potential Use of Extracellular Vesicles

**FIGURE 3:**
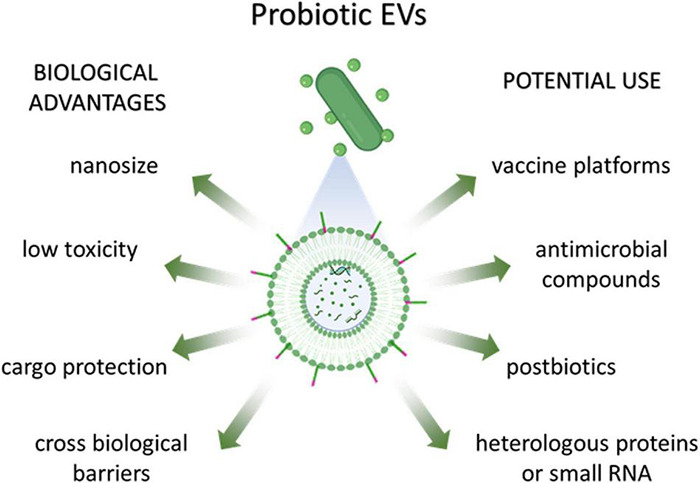
Biological advantages and potential use of probiotic-derived EVs.

The use of EVs as delivery systems could provide several advantages including their nanosize, their biocompatibility in comparison to synthetic drug delivery systems (low toxicity), the ability to cross biological barriers, their ability to protect their cargo from unfavorable environmental conditions (pH, enzymes, oxidative stress) and the possibility of engineering parent cells to modify EV composition (Figure 3). There is still a huge gap between basic research and clinical trials as far as bacterial EVs are concerned.

Postbiotics are a novel clinical strategy to consider for the treatment of infections in absence of cells. For example, in diabetic foot ulcers, skin barrier is impaired and thus administration of live bacteria is not a safe approach (Nam et al., 2021). Here is where probiotic-derived EV administration could fall into place and replace probiotic beneficial effects like pathogen inhibition and immunomodulation.

To prevent infectious diseases, only Gram (−) pathogenic bacterial EVs have been used as vaccines up to now, showing to be safe and efficacious on several occasions, while others are under evaluation (Behrens et al., 2021). For example, there are clinically available EV-based vaccines against *Neisseria meningitidis*, a causative agent of meningitis. The development of EV-based vaccines is a promising field for the prevention of infections. However, the isolation of EVs from several pathogenic microorganisms for vaccine design may have limitations. For example, many pathogens like bacteria, fungi and parasites cannot be cultured in the laboratory (Li et al., 2014; Roig et al., 2018). In the case of viruses, which do not produce EVs, cell cultures are necessary for the design of EV-based vaccines (Shehata et al., 2019; Yang et al., 2021). In this line, vaccination with engineered EVs from probiotic bacteria could be a useful platform to express pathogen antigens to be used as vaccines without toxicity in humans. To our knowledge, *E. coli* Nissle 1917 was the only strain assessed for this application in an animal model (Rosenthal et al., 2014). Further studies comparing Gram (−) and Gram (+) probiotic EVs would be necessary to elucidate whether the presence of certain components like LPS or LTA on the surface is important for the enhancement of the immune response. It is important to highlight that different chemical composition of LPS and LTA induce differential inflammatory responses and this must be taken into account to enhance EV immunogenicity (Migale et al., 2015; Jastrząb et al., 2021).

On the other hand, to treat infectious diseases, genetic engineering could be exploited for pathogen inhibition by increasing the expression of antimicrobial peptides and further encapsulation in EVs (Dean et al., 2020). Bacteriocins are potent small antimicrobial peptides synthesized by certain bacteria that may be appointed as alternatives to traditional antibiotics (Gradisteanu Pircalabioru et al., 2021). Bacteriocins within EVs turn them into potential candidates against infections, including those caused by antimicrobial resistant pathogens. According to WHO, antimicrobial resistance continues to be a global health and development threat (World Health Organization, 2021). Indeed, an important advantage of probiotic administration is the reduction in the use of strong anti-inflammatory agents and/or antibiotics that can be unfavorable in the long term (Kasatpibal et al., 2017; Guo and Leung, 2020; Raheem et al., 2021). In this regard, the indiscriminate use of antimicrobials leads not only to the development of antimicrobial resistance in pathogens, but also to the loss of our microbiota. The latter increases the susceptibility to infections such as vaginal candidiasis (Xu et al., 2008). Administration of probiotic EVs could be used not only to treat and/or prevent infections, but also would decrease antimicrobial use.

By taking advantage of EV versatility, other genetic engineering approaches can be applied to modify EV cargo or surface for the delivery of drugs to target cells. Genetic engineering enables the overexpression of proteins or the synthesis of small RNA that could silence target host genes (Fantappiè et al., 2014; Koeppen et al., 2016). EV cargo could be protected from harsh environmental conditions and additionally surface molecules could direct EVs to target host cells. This strategy could be relevant for the delivery of two or more synergistic drugs and/or the delivery of compounds that have difficulties in crossing the cell membrane (Liu et al., 2018).

### Missing Knowledge and Challenges

As documented in several studies, probiotic-derived EVs could be involved in the prevention and treatment of infectious diseases. However, the protective capacity of probiotic bacteria EVs against pathogen infections was only studied against one virus (HIV-1) and a few bacteria (*S. aureus, S. typhimurium* and *E. coli*) (Mata Forsberg et al., 2019; Ñahui Palomino et al., 2019; Hu et al., 2020). Therefore, there is still no information on its beneficial effect against fungal and parasitic infections.

To date, there are many unknowns regarding the use of probiotics EVs as pharmaceutical agents. Current challenges are the lack of standardized and cost-effective methods for EV isolation, purification, characterization and upscale processing (Gurunathan et al., 2021). Unlike human EV markers, specific bacterial EV markers remain mostly unidentified (Ñahui Palomino et al., 2021). Identifying these molecular markers could not only optimize current characterization techniques, but also improve our understanding about EV physiology and future possible biomedical applications. For example, the probiotic *B. subtilis* produced *S. aureus* intestinal decolonization by inhibiting the pathogen quorum-sensing, and thereby produced a general decolonization (including the nose) (Piewngam and Otto, 2020). It would be interesting to study if probiotic EV components can mediate the inhibition of quorum-sensing among pathogenic bacteria. Even more, advances in the understanding of the role of EVs in inter-kingdom communication will almost certainly provide valuable insights into the development of novel therapies against pathogens.

Regarding the use of probiotic bacteria to create engineered EVs with vaccination purposes, the expression of antigenic proteins from non-culturable eukaryotic pathogens (fungi and parasites) has some limitations related to bacterial ability to make post-translational modifications. In this case, expression of antigens in eukaryotic probiotic organisms like yeasts would be a better and low cost option.

One alternative to administering isolated EVs that remains to be evaluated is whether it would be more advantageous to administer functional food with probiotics as a platform for EV delivery. As far as we know, EVs are constantly secreted by metabolically active bacteria (Brown et al., 2015; Liu et al., 2018). In a bacterial culture, EV release can vary depending on the growth conditions, including pH, oxygen presence, and agitation rate (Müller et al., 2021). For example, at pH 5 *L. plantarum* released a smaller number of EVs than at pH 7. On the other hand, there is recent evidence that *L. rhamnosus* JB-1 EVs can reach the bloodstream of mice after oral administration of the probiotic (Champagne-Jorgensen et al., 2021a). This outcome strongly suggests *in situ* EV release in the GIT. Whole cells would resist better than EVs to conditions during storage and transit through the GIT. In the case of spore-producing probiotics (e.g., *B. subtilis*), spore administration would be a cost-effective option. In this way, problems concerning EV stability would be avoided. Another strategy to consider is the microencapsulation of probiotics contained in food matrices to improve their viability during storage and in the GIT (Qi et al., 2020). Besides, if the encapsulating agent is mucoadhesive, a longer residence time in the GIT may allow a sustained release of EVs over time (Yao et al., 2020). Another microparticle-based delivery system could be a particle with coupled EVs on the surface to achieve high concentrations of EVs, maximizing EV effects as demonstrated in *in vitro* models (Kuhn et al., 2020).

## Conclusion

The new era of postbiotics has brought a new point of view on the beneficial effects of probiotics. Probiotic-derived EVs could be mediating, at least in part, the beneficial effects of probiotics against infectious diseases via: inhibition of pathogens, enhancement of epithelial barrier function and modulation of the immune system. Remarkably, EVs can reach the bloodstream and consequently be delivered to extraintestinal organs, where probiotics were shown to have beneficial effects. Future studies should be focused on the characterization of EV active components and their interaction with the host. Novel EV-based technologies are promising for the design of therapies and/or vaccines against infections. Moreover, probiotics contained in food matrices could be used as EV-releasing devices in the GIT with potential applications in the functional food industry.

## Author Contributions

APDR and CLD designed the idea, collected literature data, created the tables and figures, and wrote the manuscript. MP and OP reviewed and approved the final version of the manuscript. All authors contributed to the article and approved the submitted version.

## Conflict of Interest

The authors declare that the research was conducted in the absence of any commercial or financial relationships that could be construed as a potential conflict of interest.

## Publisher’s Note

All claims expressed in this article are solely those of the authors and do not necessarily represent those of their affiliated organizations, or those of the publisher, the editors and the reviewers. Any product that may be evaluated in this article, or claim that may be made by its manufacturer, is not guaranteed or endorsed by the publisher.
